# Peripubertal stress increases play fighting at adolescence and modulates nucleus accumbens CB1 receptor expression and mitochondrial function in the amygdala

**DOI:** 10.1038/s41398-018-0215-6

**Published:** 2018-08-15

**Authors:** Aurélie Papilloud, Isabelle Guillot de Suduiraut, Olivia Zanoletti, Jocelyn Grosse, Carmen Sandi

**Affiliations:** Laboratory of Behavioral Genetics, Brain Mind Institute, School of Life Sciences, Ecole Polytechnique Fédérale de Lausanne, Lausanne, Switzerland

## Abstract

Play fighting is a highly rewarding behavior that helps individuals to develop social skills. Early-life stress has been shown to alter play fighting in rats and hamsters as well as to increase aggressive behaviors at adulthood. However, it is not known whether individual differences in stress-induced play fighting are related to differential developmental trajectories towards adult aggression. To address this question, we used a rat model of peripubertal stress (PPS)-induced psychopathology that involves increased aggression at adulthood. We report that, indeed, PPS leads to enhanced play fighting at adolescence. Using a stratification approach, we identify individuals with heightened levels of play fighting as the ones that show abnormal forms of aggression at adulthood. These animals showed as well a rapid habituation of their corticosterone responsiveness to repeated stressor exposure at peripuberty. They also showed a striking increase in mitochondrial function in the amygdala—but not nucleus accumbens—when tested ex vivo. Conversely, low, but not high players, displayed increased expression of the CB1 cannabinoid receptor in the nucleus accumbens shell. Our results highlight adolescence as a potential critical period in which aberrant play fighting is linked to the emergence of adult aggression. They also point at brain energy metabolism during adolescence as a possible target to prevent adult aggression.

## Introduction

Play fighting, also called social or rough-and-tumble play, is a common form of play observed in many species, such as mammals and birds. It is typically most abundant from weaning until puberty, with a peak at adolescence (32 to 40 days of age in rodents) and gradually declines with the approach of sexual maturity^1^. Play fighting facilitates the development of cognitive, emotional, and social skills, and the ability to use these skills when confronted with an unpredictable situation later in life^2–4^.

The peripuberty period, comprising childhood and adolescence, is a critical time window in brain development, sensitive to deleterious effects of adverse experiences. Several studies have reported enhanced risk of developing psychopathologies later in life following stress exposure at early life, in both humans and animals^5–7^. Importantly, social play has been shown to be modulated by early life experiences. Thus, maternal separation during the first 2 weeks of life resulted in increased play fighting in male rats^8^; however, the opposite effects were described for mice^9^. Likewise, juvenile restraint stress for 5 days^10^ and post-weaning social isolation for a period of 4 weeks^11^ led to increased play fighting in rats. In hamsters, juveniles exposed daily from P28 to P42 to an aggressive adult male showed premature transition from play fighting to adult-like attack, and increased aggression at adulthood^12^. Our lab has developed an animal model of psychopathology based on stress at peripuberty, in which rats are exposed to fearful stimuli on a basis of seven non-consecutive days from postnatal day 28 to 42. Previous work has demonstrated deficits in social behaviors, including reduced sociability and heightened aggression, in peripubertally stressed adult rats^13–16^. Interestingly, corticosterone administration at the exact schedule as our peripubertal stress (PPS) model mimicked alterations in the social domain and led as well to enhanced play fighting^17^. However, it is not known whether PPS affects play behavior and, therefore, in this study, we aimed to investigate the consequences of PPS on play fighting and examined neurobiological correlates.

Play fighting is a highly rewarding behavior modulated by neural systems that are implicated in reward processing, including endocannabinoids, opioids, and dopamine^4,18–20^. In the brain, the endocannabinoid system works principally through the G-coupled cannabinoid receptor 1 (CB1). The endocannabinoid system has been implicated in a myriad of functions, including the regulation of the HPA axis^21,22^ and play behavior. Systemic injection of an endocannabinoid agonist was shown to increase play fighting though actions on the CB1 receptor, and the nucleus accumbens (NAc) and basolateral amygdala (BLA) have been implicated in these effects^23,24^. Importantly, CB1 receptors have been shown to be expressed in the mitochondrial membrane, where they act on the regulation of energy metabolism and mitochondrial functions^25–27^.

Here, we first asked whether PPS affects play fighting during adolescence and, then, applied a stratification approach to evaluate whether the levels of play fighting predict aggressive behavior at adulthood. In this connection, we examined whether differences in corticosterone responsiveness to repeated exposure to stressors at peripuberty are linked to differences in play fighting. Finally, we explored the modulation of neurobiological mechanisms in the BLA and NAc by PPS and their potential different engagement in animals with different levels of play fighting. Specifically, we focused in the endocannabinoid system and mitochondrial function.

## Materials and methods

### Animals

Subjects were the offspring of Wistar Han rats (Charles River Laboratories, France), bred in our animal facility. Rats were maintained under standard housing conditions on a 12 h light-dark cycle (lights on at 7:00 am). Food and water were available ad libitum. All experiments were performed with the approval of the Cantonal Veterinary Authorities (Vaud, Switzerland) and carried out in accordance with the European Communities Council Directives of 24 November 1986 (86/609EEC).

### Experimental design

At weaning, male rats from different litters were randomly assigned to control (CTRL) and PPS conditions. They were distributed into home cages in dyads of two non-siblings. On postnatal day 28 (P28), the PPS protocol began (see below). This study included three experiments. In experiment 1, CTRL and PPS rats underwent play fighting test 3 days after the end of the PPS protocol (i.e. P45), followed by three behavioral tests for sociability (social preference), aggression (resident-intruder), and depressive-like behavior (forced-swim test) at adulthood (see Supplementary Materials and Methods for further details on the tests). In experiment 2, CTRL and PPS rats underwent play fighting test after the PPS protocol and were killed 24 h later for molecular analyses. In the last experiment, CTRL and PPS rats were sacrificed directly at the end of the play fighting test to proceed to the measurements of mitochondrial respiration. Animals were killed 24 h after play fighting for gene analyses; directly after play fighting for mitochondrial respiration analyses; and 3 days after the last behavior when tested at adulthood. The sample size was calculated following previous studies from our lab with the PPS model and estimated to be between 8 and 12. The sample size for each experimental group is indicated in the legend of the figure. The experimenter was blind during both testing and analysis.

### Peripubertal stress

The PPS protocol is based on exposure to fear-induction procedures and was described in details previously^14^. Briefly, following exposure to an open-field for 5 min on P28, the stress protocol consisted of presenting two different fear-inducing stressors, each one lasting for 25 min. They were either exposure to the synthetic fox odor trimethylthiazoline (TMT) (9 ml) (Phero Tech Inc., Canada), which was administered in a plastic box (38 × 27.5 × 31 cm) or to an elevated platform (EP) (12 × 12 cm), and were presented either alone or in combination. Following each stress session, the animals remained separated for 15 min before rejoining their cage mates. The stressors were applied during the peripuberty period (a total of 7 days across P28 to P42) during the light phase and followed a variable schedule. CTRL animals were handled on the days that their PPS counterparts were exposed to stress. Animals in the same cage were always assigned to the same experimental group (either CTRL or PPS).

### Play fighting test

The play fighting test was performed on the days immediately after the end of the PPS following a protocol adapted from Trezza and colleagues^28^. On P43 and P44, rats were individually habituated to the testing cage for 10 min. On P45, animals were socially isolated for 3.5 h before testing, to enhance their social motivation. The test consisted of placing two animals from the same experimental group, though not familiar to each other, into the test cage for 15 min. The animals on each dyadic encounter did not differ >10 g in body weight. The interaction was video-recorded and behaviors were manually scored offline by a trained experimenter blind to experimental groups, which was assisted by the Observer software (Noldus IT; Netherlands). Duration of the following behaviors was quantified: pinning (i.e. keeping down), pouncing (i.e. rubbing the neck), boxing, kicking, and social exploration. The cumulative duration of pinning, pouncing, boxing, and kicking was summed to provide a measure of total play fighting. The latency to play fight was also recorded.

Stratification of animals’ behavior was performed to classify animals for differences in fighting behaviors. Classification criteria were defined according to the extremes (20th or 80th percentile, depending on index) of the CTRL group’s distribution for each measure, including total duration of play fighting, latency to play fight, frequency of play fighting, percentage of pinning, and percentage of pouncing. Every rat score above (or below in the case of the sole 20th percentile measure: latency to play fight) the cutoff for a particular measure was scored as being ‘high player’ in that measure. When a rat accrued three such scores, out of a possible of five, it was considered as a ‘high player’ rat overall.

### Resident-intruder test

The resident-intruder protocol was adapted from Veenema et al.^29^ and performed following the same conditions as those employed in the previous studies from our laboratory^14^. Briefly, prior to the night of the test, experimental rats cohabited with a female partner for 10 days, in order to encourage territoriality. The female was removed 30 min before the onset of the test and replaced afterwards. The test was conducted during the dark cycle. The resident was exposed in its home cage to a smaller (~ 5–10% lighter), unfamiliar male intruder of the same strain for 30 min. Each intruder was used only once. The following parameters were quantified in terms of duration: attack, offensive upright, keeping down, lateral threat, social investigation, and auto-grooming. The cumulative duration of the attack, offensive upright, keeping down, and lateral threat were summed to provide a measure of total offensive behavior. The latency to first offense and the frequency of abnormal attacks were also quantified. Aggressive behaviors can be both ‘normal’ (i.e. within species-typical norms) and ‘abnormal’ in nature^30^. As described by Haller^30^, we considered abnormal forms of aggression to include attacks that were excessively violent (i.e. causing a strong reaction in the bitten rat), unsignaled or targeted towards vulnerable body parts.

### Corticosterone measurement

On P28, P30, and P42 blood samples from PPS animals were collected by tail-nick (100–150 µl). The sampling was done immediately after stress offset and then 30 min after the first blood sampling. During this interval, rats were placed in a novel cage and were prevented of direct physical contact with their cage mates. After the second blood sampling, animals were placed back in their home cage.

### Gene expression analysis

Rats were decapitated in the morning (from 9:00 until 11:30 am) under basal conditions on P46. After decapitation, the brains were snap frozen in isopentane at −45 °C and stored at −80 °C. They were then sectioned using a cryostat and 200 µm thick slices were mounted on slides in order to punch and remove the region of interest, with puncher of appropriate diameter. The tissue was collected in RNAse-free tubes. Total RNA from the regions of interest was isolated using the RNAqueous Micro kit (Ambion, Life Technologies, USA), and complementary DNA was synthesized using the qScript cDNA Mastermix kit (Quanta Biosciences, USA) according to supplier’s recommendations. For real-time quantitative polymerase chain reaction (qPCR), reactions were performed in triplicate using SYBR Green PCR Master Mix (Applied Biosystems, Life Technologies, USA) in an ABI Prism 7900 Sequence Detection system (Applied Biosystems, Singapore). Two genes were used as internal controls: actin gamma 1 (ActG1) and eukaryotic elongation factor 1 (EEF1). We analyzed the expression of the CB1. Additionally, we assessed the expression of genes involved in the mitochondrial function from the homogenates used for mitochondrial respiration measurement (see Supplementary Materials and Methods). Primers for the genes of interest were designed using the Assay Design Center software from Roche Applied Science (see Table S1 in Supplementary Materials and Methods). Gene expression was analyzed with the qBase 1.3.5 software using the comparative cycle threshold methods.

### Mitochondrial respirometry

Rats were killed by rapid decapitation on P45, directly at the end of the play fighting test. The samples were processed as previously described for other tissues^31^. A multisubstrate protocol was used to sequentially exploring the various components of mitochondrial respiratory capacity, as previously described for the NAc and BLA^32,33^. See Supplementary Information for further details.

### Statistical analysis

Data were analyzed with two-sided Student *t*-test, ANOVAs and repeated measures ANOVAs, as appropriate, using GraphPad Prism 6 (GraphPad software Inc., USA). Post hoc analyses were performed with Bonferroni tests. If one animal was considered as outlier, defined as more than three deviations from the mean, it was excluded from statistical analysis. For the *t-*test, if Levene’s test for equality of variances was significant, equal variance was not assumed and the altered degree of freedom was rounded to the nearest whole number. In case of violation of the assumptions required to analyze stratification according to play fighting level, Kruskal–Wallis test was used instead of one-way ANOVA, followed by Dunnett’s post hoc test. Data from the mitochondrial respiration were analyzed using SPSS statistical software version 17.0 (SPSS, Chicago, IL). As experiments were performed in blocks across days, a linear mixed model was created that included block as a random effect in addition to fixed effects of stress or play fighting level. The estimated marginal means of the model are then reported. Pearson correlation coefficient (r) was calculated to establish relationships between CI- and CII-dependent respiration and play fighting percentage or gene expression in amygdala and NAc homogenates. All bars and error bars represent the mean ± SEM. Significance was set at *p* < 0.05, while the *p*-values were considered tending toward significance when 0.05 ≤ *p* ≤ 0.1. Graphs were created using GraphPad Prism 6.

For further information on the social preference and forced swim tests, gene expression and mitochondrial respirometry analyses, see Supplementary Materials and Methods.

## Results

### Exposure to PPS leads to enhanced play fighting

**Fig. 1:**
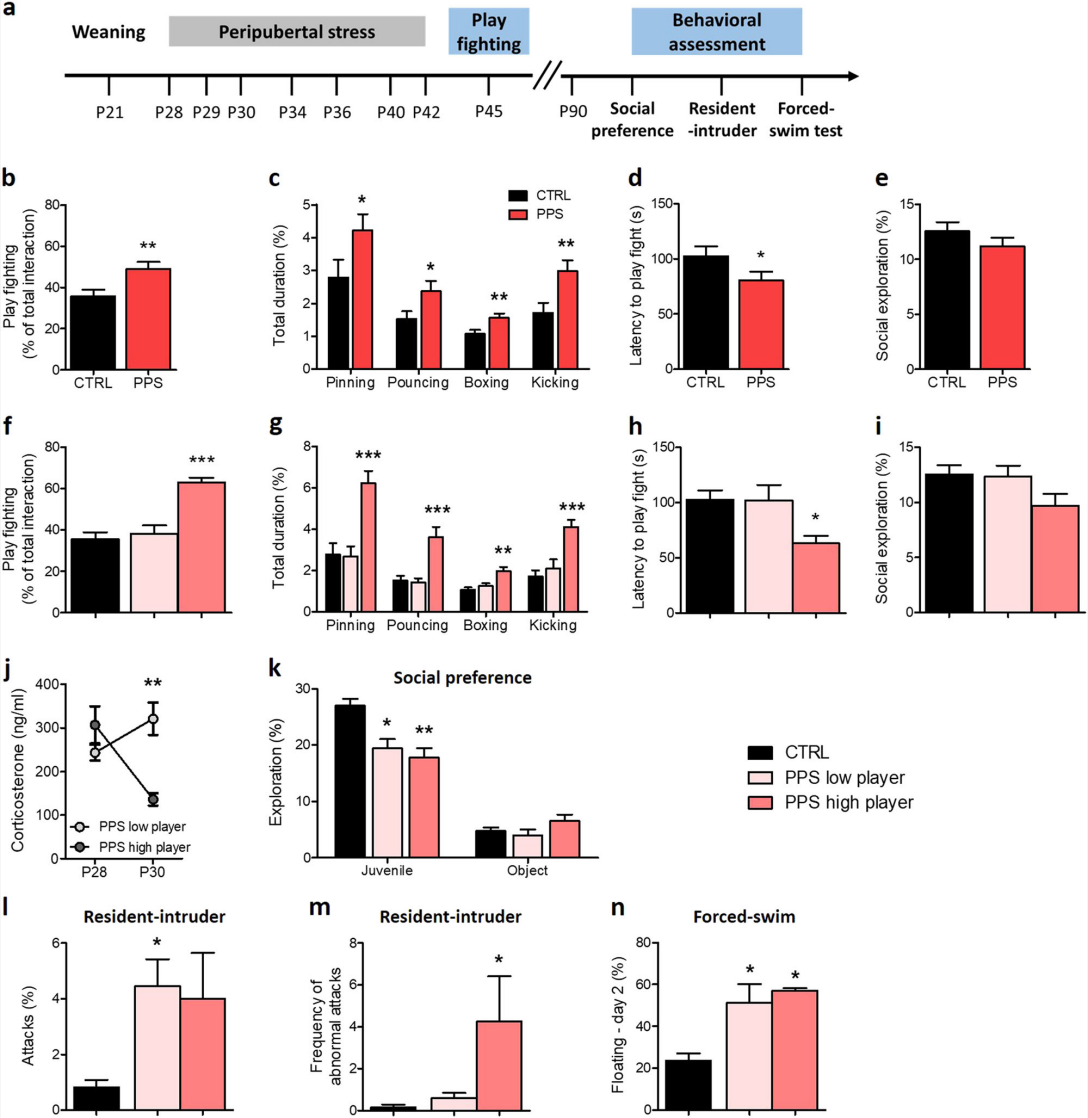
Effects of peripubertal stress on play fighting, corticosterone levels and behavioral analysis according to play levels. Timeline of the experiment (**a**). Rats stressed at peripuberty (PPS) showed higher play fighting than control (CTRL) animals (**b**). The duration of pinning, pouncing, boxing, and kicking was also increased in PPS rats (**c**). Furthermore the latency to play fight was shorter in stressed animals compared to CTRLs (**d**). No significant difference was found in the social exploration (**e**). PPS high player group exhibited higher play fighting than CTRL and PPS low players (**f**). They also exhibited increased pinning, pouncing, boxing, and kicking duration (**g**) and decreased latency to play fight (**h**). No significant change was observed in the social exploration (**i**). Corticosterone analysis revealed similar plasma levels on P28 between high and low players, whereas PPS high player rats showed lower corticosterone levels than low players on P30 (**j**). At adulthood, in the social preference test, both PPS high and PPS low players exhibited decreased juvenile exploration, but the object exploration was similar between groups (**k**). In the resident-intruder test, PPS low players showed more attacks than CTRL rats (**l**). The frequency of abnormal frequency was higher in PPS high player rats compared to CTRLs (**m**). In the forced-swim test, both PPS low and PPS high players spent more time floating in the second day of the test (**n**). *N:* CTRL = 8 or 17; PPS low player = 5–7; PPS high player = 5 or 10, except for play fighting data, where CTRL = 29; PPS = 30; PPS low player = 13, and PPS high player = 17. *p < 0.05, **p < 0.01, ***p < 0.001, vs CTRL and/or vs PPS low players. Results are expressed as mean ± SEM

First, we asked whether exposure to PPS modifies play behavior during adolescence, in an experiment performed at P45 (Fig. 1a). We found that, indeed, PPS rats showed significantly higher play fighting than CTRL animals, both for the compound index (Fig. 1b; *U* = 252.00, *p* < 0.01) and, individually, for each of the respective play fighting behaviors (pinning: *U* = 269.50, *p* < 0.05; pouncing: *U* = 284.00, *p* < 0.05; boxing: *U* = 243.00, *p* *<* 0.01; kicking: *U* *=* 247.50, *p* *<* 0.01; Fig. 1c). PPS rats also exhibited shorter latency to play fight than CTRLs (Fig. 1d; *U* *=* 270.00, *p* < 0.05). However, the two groups did not differ in social exploration during the encounter (Fig. 1e; *t*_56_ = 1.19, n.s.).

Then, PPS animals were stratified according to their level of play fighting as either high or low player (see Materials and Methods) (Fig. 1f; *H*(3) = 21.70, *p* *<* 0.001). Following this approach, we found that whereas play levels in the PPS low player group were similar to CTRLs, in the PPS high player group they were significantly higher than both CTRL and PPS low player animals (all, *p* < 0.001). The same picture was observed when each play fighting parameter was evaluated independently (pinning: *F*_(2, 57)_ = 9.80, *p* < 0.001; pouncing: *H*(3) = 18.49, *p* < 0.001; boxing; *H*(3) = 12.67, *p* < 0.01; kicking; *H*(3) = 16.59, *p* < 0.001, post hoc analyses *p* *<* 0.01 or *p* < 0.001; Fig. 1g). The latency to play fight was also different between the three groups (Fig. 1h; *H*(3) = 6.52, *p* < 0.05) due to a shorter latency in PPS high player rats than CTRLs (*p* < 0.05). Again, no significant differences were observed between groups in social exploration (Fig. 1i; *F*_(2, 57)_ = 2.16, n.s.).

We also analyzed whether the high and low player groups differed in their corticosterone responses to stressor exposure on days 1 and 3 (i.e., P28 and P30, respectively). We found that whereas plasma corticosterone levels did not differ for the two groups following the first exposure to stress (P28) (Fig. 1j; *U* = 4.00, n.s.;), the high player group showed markedly lower corticosterone levels than the low player group following stressor exposure to the third day (P30) (Fig. 1j; *U* = 0.00, *p* *<* 0.01).

### Peripubertally stressed rats that show high play fighting at adolescence display abnormal aggressive behaviors at adulthood

Then, we examined whether differences in play fighting are associated with vulnerability to display alterations in a number of behaviors at adulthood—such as sociability, aggression, and stress coping behaviors (Fig. 1a)—known to be modified in peripubertally stressed rats^14–16,34,35,36^. In the social preference test, PPS induced a decrease in the exploration of the juvenile as compared to CTRLs (Fig. 1k; *H*(3) = 16.20, *p* < 0.001). However, both PPS low (*p* < 0.05) and high player (*p* < 0.001) groups exhibited reduced exploration but did not differ between themselves. No significant change was observed in the time spent exploring the object (Fig. 1k; *H*(3) = 2.49, n.s.).

In the resident-intruder test, ANOVA for attack percentage was significant (Fig. 1l; *H*(3) = 6.71, *p* < 0.05), with post-tests revealing an increase in attacks in PPS low players compared to CTRL rats (*p* < 0.05). However, PPS high player animals showed increased frequency of abnormal attacks compared to CTRLs (Fig. 1m; *H*(3) = 6.06, *p* < 0.05; post hoc analyses *p* < 0.05). The latency to first offense was similar between groups (*H*(3) = 2.31; data not shown). Interestingly, we found positive correlations between play fighting parameters at adolescence (i.e., total play fighting, boxing) and specific types of aggressive behaviors at adulthood (i.e., keeping down, abnormal attacks) (Figure S1a–c).

In the forced-swim test, although floating time on the first day did not differ between groups (*H*(3) = 3.02, n.s., data not shown), peripuberty stress had an effect on the second day of testing (Fig. 1n; *H*(3) = 10.05, *p* < 0.01). Both PPS low and high players showed higher floating time than CTRLs (*p* < 0.05).

### PPS affects CB1 gene expression in the NAc shell

**Fig. 2:**
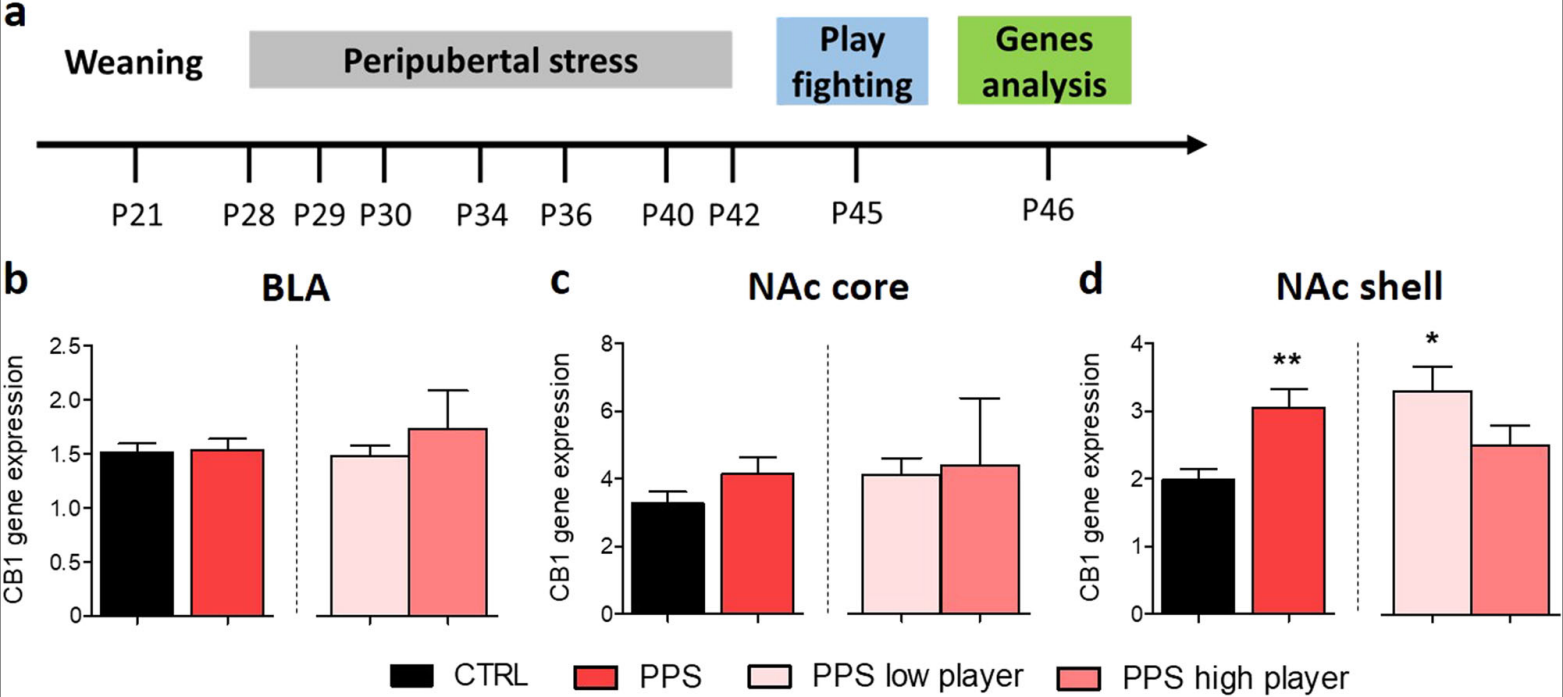
Analysis of CB1 gene expression following play fighting in the BLA (left), NAc core (middle) and NAc shell (right). In the basolateral amygdala (BLA), the expression levels of CB1 were not found to be different between groups (**b**). In the NAc core, the expression was also similar among groups (**c**). CB1 expression in the NAc shell was increased in PPS rats compared to CTRL animals, whereas PPS low players have increased CB1 expression compared to CTRL (**d**). *N:* CTRL = 10–12 and PPS = 9–11; PPS low player = 8, PPS high player = 3. *p < 0.05, **p < 0.01, vs CTRL. Results are expressed as mean ± SEM

Twenty-four hours after the play fighting test, samples were taken for gene expression analyses to quantify the CB1 in the BLA and in the NAc shell and core. No group differences in gene expression were found in the BLA (Fig. 2b; *t*_20_ = 0.12, n.s., even when just the two PPS groups were compared: all *p* > 0.05). Similarly, in the NAc core, no significant differences between groups were observed (Fig. 2c; *t*_18_ = 1.45, n.s. even when just the two PPS groups were compared: all *p* > 0.05). In the NAc shell, CB1 expression levels were higher in PPS animals than in CTRLs (Fig. 2d, *t*_20_ = 3.27, *p* < 0.01), and PPS low players showed higher CB1 expression than CTRLs (*H*(3) = 8.50, *p* < 0.01, post hoc analyses *p* < 0.05; Fig. 2d). Furthermore, when controls were as well stratified as either high or low players following the same criteria as for PPS rats, we found no difference in CB1 expression in none of the three brain regions analyzed [Figure S2a-c; but note that CTRL high players do not show abnormal attacks (Figure S2d) and statistics are not possible due to the low number (*n* = 2) of high players in this group]. We also performed correlational analyses and obtained no evidence to support a link between CB1 expression levels in each of the brain regions in PPS animals and the level of play fighting behaviors (Figure S3). Additional analyses failed to identify PPS-related changes in the expression levels of fatty acid amide hydrolase (FAAH) and monoacylglycerol lipase (MAGL), two catabolic enzymes of the endogenous ligands AEA and 2-AG, in these three brain regions (see Supplementary Figure S4). However, a note of caution should be added regarding these observations: FAAH and MAGL are enzymes whose biological effects can be modified at the level of activity; therefore, lack of stress-induced differences in their mRNA content cannot be interpreted as absence of stress effects in their function. Furthermore, we tested whether differences in CB1 expression could be observed in adult rats submitted to PPS and classified according to play fighting levels at adolescence. While we did not find differences in the amygdala or in the NAc shell, CB1 expression levels in the NAc core were significantly lower in PPS low than in high players (Figure S5).

### PPS leads to enhanced mitochondrial function in the amygdala following play fighting

**Fig. 3:**
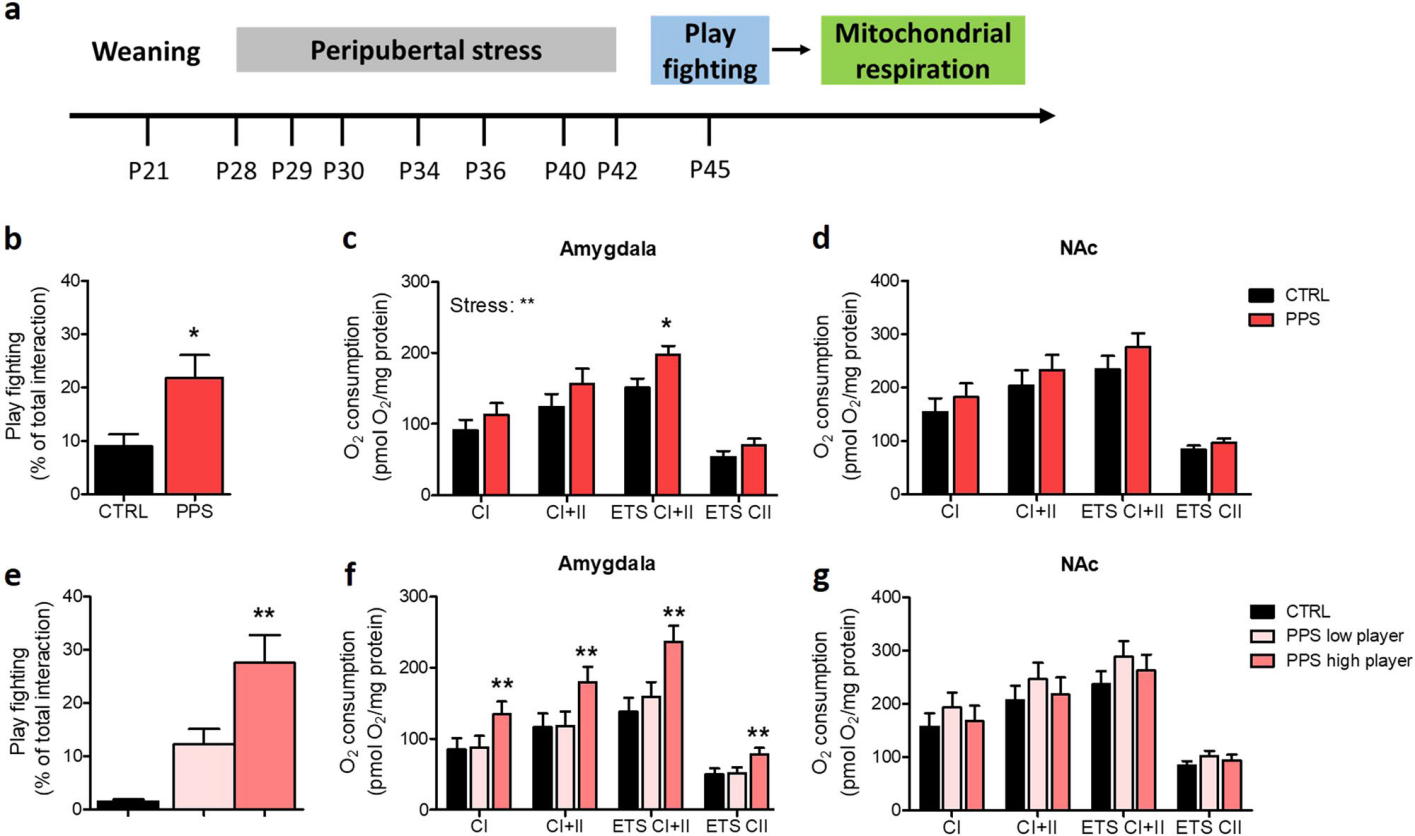
Mitochondrial respiration following play fighting. Timeline of the experiment (**a**). Play fighting was increased in PPS rats compared to CTRLs (**b**). A main effect of stress was found in the mitochondrial respiration in the amygdala and the maximal electron transfer system capacity being significantly higher in PPS rats than CTRLs (**c**). No significant change was observed in the mitochondrial respiration in the nucleus accumbens (NAc) (**d**). Analysis according to play fighting level confirmed that PPS high players showed higher play fighting than to CTRLs (**e**). Moreover, amygdala respiration was increased in PPS high players compared to CTRLs and PPS low players (**f**), whereas no significant change was observed in the respiration in the NAc (**g**). *N:* CTRL = 8, PPS = 8, PPS low player = 3, and PPS high player = 5. *p < 0.05, **p < 0.01, vs CTRL, and also vs PPS low player in (**e**). Results are expressed as mean ± SEM. Respiration data are presented as estimated marginal means ± SEM of oxygen flux per mg tissue

**Fig. 4:**
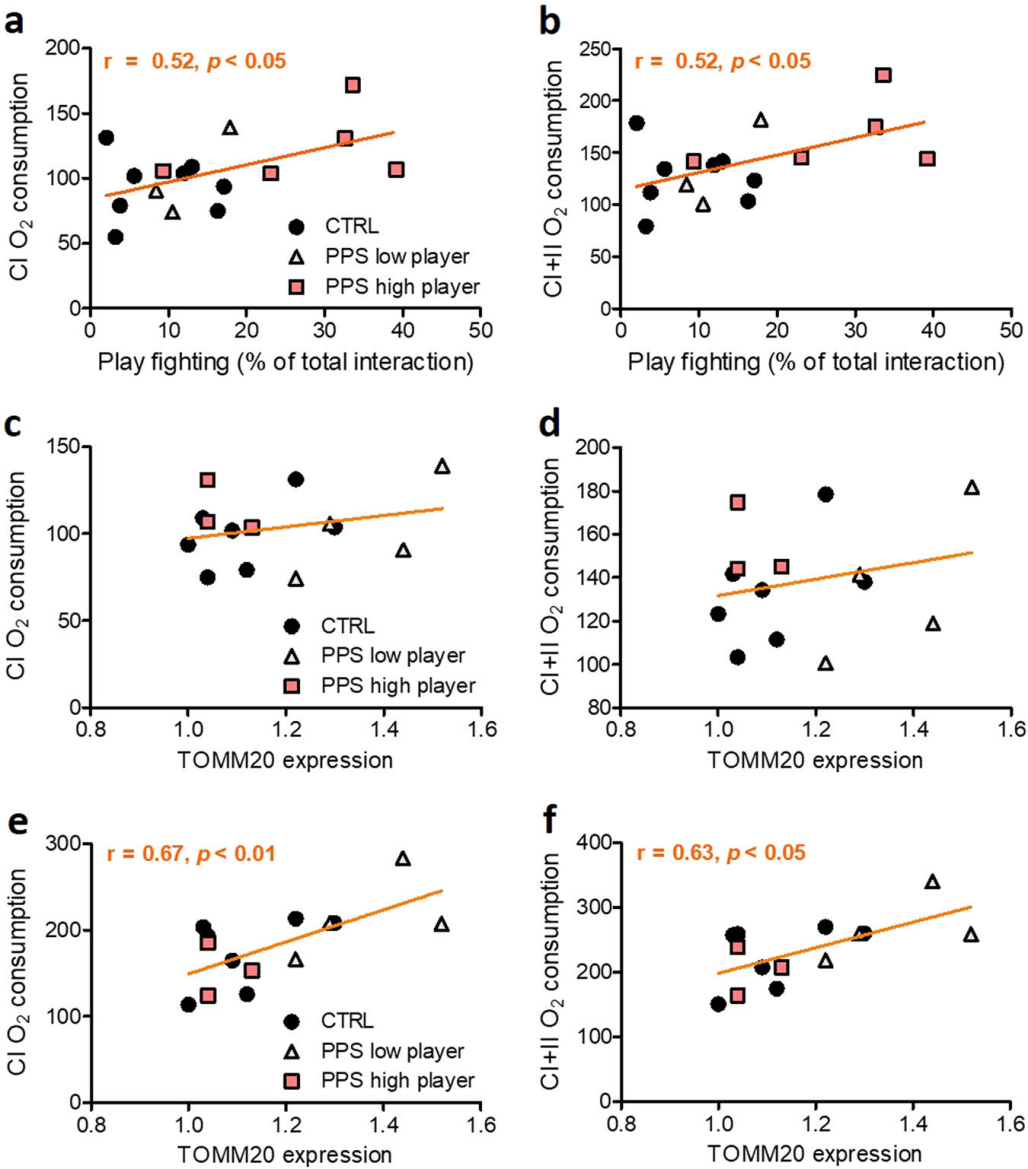
Correlations between respirometry parameters and play fighting or gene expression in the amygdala or NAc. Play fighting positively correlated with complex I- and complex II-dependent respiration measured in the amygdala, when taking into account all data together (**a**, **b**). Gene expression of TOMM20 in the amygdala homogenates did not correlate with respirometry parameters from amygdala measurement (**c**, **d**), whereas it positively correlated with nucleus accumbens (NAc) data (**e**, **f**). *N:* CTRL = 8, PPS low player = 3, and PPS high player = 5. Results are expressed as mean ± SEM

In a subsequent experiment, we measured mitochondrial respiration at the offset of the play fighting test (Fig. 3a). We confirmed that both PPS effect as a whole (Fig. 3b; *U* = 12.00, *p* < 0.05), and PPS high player rats (Fig. 3e; *H*(3) = 12.01, *p* < 0.01, followed by post hoc test: *p* < 0.01) showed enhanced play fighting compared to CTRL animals. Strikingly, a main effect of stress was found in ex vivo measurements of mitochondrial respiration in the amygdala (Fig. 3c; *F*_(1, 56)_ = 7.56, *p* < 0.01) but not in the NAc (Fig. 3d; *F*_(1, 56)_ = 2.86, n.s.). The maximal electron transport system capacity (ETS) was also increased in the amygdala of PPS animals (*p* *<* 0.05). Analyses based on the stratification for play fighting levels revealed that PPS high player individuals exhibited higher mitochondrial respiration in each of the mitochondrial parameters analyzed than CTRL and PPS low player rats (all *p* < 0.01) in the amygdala (Fig. 3f). No significant differences in the mitochondrial respiration in the NAc were found according to this analysis (Fig. 3g). Interestingly, we found significant positive correlations between play fighting percentage and respiration in amygdala (CI: *r*_16_ = 0.52, *p* < 0.05; CI+II: *r*_16_ = 0.52, *p* < 0.05) (Fig. 4a, b). Complex I- (Fig. 4e) and complex II-dependent (Fig. 4f) respiration also correlated positively with expression levels of the structural mitochondrial protein TOMM20 in the NAc (CI: *r*_14_ = 0.67, *p* < 0.01; CI+II: *r*_14_ = 0.63, *p* < 0.05), but not in the amygdala (Fig. 4c and d; respectively) (CI: *r*_14_ = 0.26, n.s.; CI+II: *r*_14_ = 0.24, n.s.). When data from controls was analyzed according to the same stratification based on high and low players, no differences in mitochondrial function were observed in the amygdala or the nucleus accumbens [Figure S2e,f; but note that statistics are not possible due to the low number (*n* = 2) of high players in CTRL animals]. Given the differences observed in mitochondrial respiration after PPS exposure, we then analyzed the expression of genes related to mitochondrial function—mitofusin 2 (MFN2), peroxisome proliferator-activated receptor coactivator-1alpha (PGC1α), sirtuin1 (SIRT1), and translocase of outer mitochondrial membrane 20 (TOMM20)—in the same amygdala samples. However, no significant differences were observed for the expression levels of any of the analyzed genes in the BLA (Figure S6) or in the central amygdala (Figure S7). Finally, given that PPS high and low player groups differed in their regulation of corticosterone responses to stress, we assessed expression levels of the glucocorticoid (GR) and mineralocorticoid receptor (MR) in the brain regions of interest, but found no evidence for a differential regulation of these genes (Figure S8).

## Discussion

Here, we show that stress exposure at peripuberty leads to enhanced play fighting at adolescence. Strikingly, although both peripubertally stressed groups show reduced sociability and increased passive coping responses at adulthood, only those individuals with particularly heightened levels of play fighting are the ones that show abnormal forms of aggression. High play fighters also show a high degree of adaptation of their corticosterone responses to repeated exposure to stressors during the peripubertal period; i.e., plasma corticosterone levels on the third day of stress exposure were substantially lower in high than in low play fighting rats. When exploring neurobiological correlates, we found an increase in CB1 gene expression in the NAc shell of peripubertally stressed rats that show low, but not in those with high play fighting levels. Interestingly, when assessing mitochondrial respiratory capacity in the amygdala immediately after testing animals for play behavior, we found a marked increase in mitochondria respiration in PPS animals, corresponding to the high, but not low, fighting rats.

Play fighting is a highly rewarding behavior that helps developing social skills for adult life^3,4,18^. Our data shows that PPS increases play fighting in rats, leading to enhanced duration of a diversity of play behaviors including pinning, pouncing boxing, and kicking. This finding is consistent with former observations in different species showing that both increased play fighting and adult aggressive behaviors are elicited by early life stress. Specifically, this was shown in (i) in a rat model of neonatal maternal separation^8,29^; (ii) in hamsters submitted to social subjugation during early puberty^12,37^; and (iii) in humans exposed to early life disturbances^38^. However, most studies so far have dealt with group effects, not considering individual differences in developmental trajectories from play fighting to adult aggression. Previously, we had reported reduced sociability, increased depression-like behaviors and increased forms of abnormal aggression following PPS in rats^13–16,34,35,36^. Here, we confirm and extend these findings by showing that rats with high and low levels of play fighting at adolescence show similar alterations in sociability (i.e., reduced social exploration) and passive depression-like behaviors (increased floating in the forced swim test) and defensive aggression (increased attack numbers in the resident-intruder test) at adulthood. Importantly, we identify stressed high players at adolescence as the ones that specifically show increased forms of abnormal behaviors. Our findings support the view that whereas the levels of play fighting do not predict total levels of adult aggressive behavior, high levels of play behavior induced by stress are associated with abnormal forms of adult aggression. We should briefly note that the stratification approach that we have followed is in line with an emerging trend in the literature, taking into account individual differences in behavioral outcomes when examining underlying neurobiology^36,39–44^.

Corticosterone analyses revealed that animals with higher levels of play fighting showed better adaptation to PPS. Given that these rats also exhibited abnormal attacks toward male intruders at adulthood and reduced sociability, these results are reminiscent of our recent observations with a line of rat that has been genetically selected for their rapid habituation to repeated stress exposure^45^. Specifically, we recently reported that constitutively high—but not low—habituating rats exposed to PPS were the only ones that developed abnormal signs of aggression^45^. In addition, these findings resonate with earlier work by Haller and collaborators in rats showing that chronic glucocorticoid deficiency induces abnormal aggression^46–48^. However, this data is at odds with recent data from children indicating a moderating role of cortisol in the association between early life stress and persistent childhood aggression, with the ELS-aggression relationship being stronger among children who had higher levels of cortisol reactivity during the preschool period^49^. However, our findings align well with evidence that a particularly severe subgroup of children with antisocial behavior show hypoactivity of the hypothalamus-pituitary-adrenal axis^50,51^.

Recent studies have emphasized a role for the endocannabinoid system in social functioning^52^ including the modulation of play fighting^53^. Given that both, intra-BLA and intra-NAc infusions of an endocannabinoid agonist increase play fighting^23,24^ through actions on the CB1 receptor^23,24,53^, we assessed CB1 mRNA levels in these brain regions. PPS led to an increase in CB1 mRNA levels in the NAc shell—but not in NAc core or in the BLA—that was particularly apparent in PPS low players. Substantial evidence supports the involvement of both NAc shell and core in play fighting. A pronounced increase in c-Fos expression has been observed in both the NAc core and shell following play fighting in rats^54^; however, only pharmacological inactivation of the NAc core, but not shell, interfered with social play behavior^55^. Furthermore, intra-NAc infusion of the mu-opioid receptor agonist DAMGO or amphetamine leads to enhanced play fighting when injected either into the core or into the shell^19,56^. Our findings showing that low, but not high, players are the ones that show upregulation of CB1 receptor in the NAc shell could, therefore, be interpreted as potential compensatory mechanisms in these animals to counteract negative effects of stress, as indexed by aberrations in the social domain.

Recently, CB1 receptors were shown to be expressed in mitochondria where they regulate energy metabolism and mitochondrial functions^25–27^. Mitochondria function and energy metabolism in the NAc was also recently implicated in social competition^32,33^ and vulnerability to social defeat^57^. Therefore, we measured mitochondrial respiration in the amygdala and NAc in CTRL and peripubertally stressed rats directly after the play behavior test. Stressed animals showed enhanced respiration in the amygdala but not in the NAc. We should note, however, that the increase in CB1 expression that we observed in the NAc of PPS rats relates to the total pool of CB1 transcripts; accordingly, no conclusion can be established regarding the effect of PPS in mitochondrial CB1 expression. In addition, given that CB1 is an axonal protein, if the expression of CB1 mRNA is changing in projection neurons, as opposed to local interneurons, then changes in CB1 receptor expression would not be locally within the accumbens shell. Therefore, there would not be a concordance between CB1 mRNA expression and potential effects in mitochondrial function in a projecting region. Given that we cannot identify the cell type in which changes in CB1 mRNA expression take place, changes in mitochondrial function related to potential interactions with CB1 might not necessarily be detected within the NAc.

Strikingly, amygdala mitochondrial respiration and play fighting percentage correlated positively. Although very little is known regarding the implication of differences in mitochondrial function in the amygdala for social behaviors, previous work showed that it does not seem to be relevant for fast fear conditioning associations^32^. Although it is tempting to speculate that the observed differences arise from behavioral differences displayed during the play behavior test given right before brain samples were taken, it should be noted that the functional mitochondrial measurements reflect maximal O_2_ consumption ex vivo when mitochondria are provided with excess substrates for each tested complex. Therefore, the measurements we took do not necessarily reflect the actual mitochondrial function in vivo before samples were taken, but mitochondrial capacity to functionally respond when electron transport complexes I and II are stimulated with substrates in excess. Given that social play fighting impinges a high metabolic cost to the organism^58^, metabolic differences arising from either early life or through the interaction with stress at puberty might be relevant in this context. In this vein, it is relevant to note that exposure to different metabolic challenges (i.e., high-fat diet, caloric restriction, exercise) during the juvenile period led to drastic changes in play behavior and expression of genes involved in bioenergetics in the brain region (prefrontal cortex) examined^59^. Another study showed that the moderate recurrent hypoglycemia during the pre-weaning period led to abnormal responses to stress and decreases in adolescent social play behavior^60^. Conversely, early postnatal overnutrition was associated with decreases in social play behavior in adolescent male rats^61^.

It is not known whether variation in mitochondrial function contributes to variation in brain region-specific activations as measured through functional MRI. In this connection, it is relevant to note that studies of children exposed to maltreatment have reported alterations in amygdala reactivity, including increased amygdala reactivity in response to angry faces^62^ and during pre-attentive emotional processing^63^ while hypoactivation to social rejection cues^64^. These children frequently display increased aggressive behaviors^65^. On the contrary, hypo-reactivity of the right amygdala to fearful faces^66^ and to theory of mind judgments^67^ was reported for children with conduct problems and elevated levels of callous-unemotional traits. Given the emerging evidence of structural and functional brain differences in children and adults who have experienced childhood maltreatment^68^ and in the context of increased aggression^69^, future studies should address the potential link between the enhanced mitochondrial function observed in our study and amygdala functionality.

A key question to address in the future is what factors and mechanisms lead to individual differences in play fighting modulation by PPS. Interestingly, a previous study showed that within-litter variation in maternal care received by individual pups correlates with adolescent social play behavior in male rats^70^. These data supports the view that subtle variations in maternal care or, supposedly, in sibling interactions might condition a different social development in male rats^70^. Alternatively, individual differences in social play fighting induced by PPS might be mediated by metabolic consequences of early life experiences (see discussion above). We should also note that an important limitation of our study is that we focused in males, while sex-specific mechanisms have been shown to regulate social play behavior^71^.

In conclusion, we report here that PPS increases play fighting, particularly in a subgroup of rats that rapidly show blunted corticosterone responses to repeated stressor exposure and display abnormal behaviors at adulthood. In addition, these animals show a drastic increase in mitochondrial respiration in the amygdala following play fighting. These results highlight adolescence as a potential critical period to prevent adult aggression by targeting heightened levels of play fighting. They open the possibility that targeting brain energy metabolism during adolescence might be a plausible avenue to prevent adult aggression.

## Disclaimer

This paper reflects only the authors’ views and the European Union is not liable for any use that may be made of the information contained therein.

## Electronic supplementary material

Supplementary Information
