# Supplementary material for: Peripubertal stress increases play fighting at adolescence and modulates nucleus accumbens CB1 receptor expression and mitochondrial function in the amygdala
Source: Transl Psychiatry. 2018 Aug 15;8:156. doi: 10.1038/s41398-018-0215-6 (PMC6093900; doi:10.1038/s41398-018-0215-6)
Supplement: Supplementary file 1 — Supplementary Information [file 41398_2018_215_MOESM1_ESM.pdf]

Supplementary information to the article:

Peripubertal stress increases play fighting at adolescence and modulates nucleus accumbens CB1 receptor expression and mitochondrial function in the amygdala

Aurélie Papilloud[a], Isabelle Guillot de Suduiraut[a], Olivia Zanoletti[a], Jocelyn Grosse[a] & Carmen Sandi[a*]

[a] Laboratory of Behavioral Genetics, Brain Mind Institute, School of Life Sciences, Ecole Polytechnique Fédérale de Lausanne, Lausanne, Switzerland

Supplementary Materials and Methods

Social preference test

The social preference test was adapted from the protocol described by Crawley and colleagues (1) to investigate social affiliation in male mice, following the same conditions previously described (14). Briefly, the test was performed in a rectangular, three-chambered box that included a central compartment and two side compartments. After five minutes of habituation to the central chamber, retractable doors were removed and the rat was allowed to explore freely the whole apparatus for 10 minutes. Side compartments were each equipped with a central, floor-fixed, transparent, perforated cylinder that contained either an unfamiliar male juvenile rat (30 ± 2 days old) or an object. The apparatus was cleaned with 5% ethanol solution and dried between each trial. The percentage of time spent exploring either the juvenile or the object was scored, and a social preference ratio was calculated according to the formula: time spent exploring the juvenile/time spent exploring the juvenile + object.

Forced-swim test

Rats were submitted to a forced-swim test to evaluate their passive-coping behavior (2), following the same conditions as already described (14). Animals were placed in a plastic beaker (25 cm diameter x 46 cm) containing 30 cm of water (25°C) for 15 minutes. A second session was performed 24h later for 5 minutes. The time spent immobile, swimming or diving was quantified.

Gene expression analysis from homogenates

Homogenates used for mitochondrial respirometry were stored in MirO5 at -80°C until RNA extraction. Samples were centrifuged 15000g for 15 minutes at 4°C before total RNA was extracted following the same protocol as described previously in Materials and Methods. The expression of the following genes was analyzed: mitofusin 2 (MFN2), peroxisome proliferator-activated receptor coactivator-1alpha (PGC1α), sirtuin1 (SIRT1) and translocase of outer mitochondrial membrane 20 (TOMM20) (Table S1)

Gene	Full name	Forward primer (5'-3')	Reverse primer (5'-3')	RefSeq (NCBI)
**ActG1**	Actin gamma 1	tagttcatgtggctcggtca	gctggggactgactgacttt	NM_001127449.1
**EEF1**	Eukaryotic translation elongation factor 1	tgtggtggaatcgacaaaag	cccaggcatacttgaaggag	NM_175838.1
**CB1**	Cannabinoid receptor 1	ggacatggagtgctttatgattc	gagggacagtacagcgatgg	NM_012784.4
**MFN2**	Mitofusin 2	attggccacaccaccaat	gctagctggttcacggt	NM_130894.4
**PGC1α**	Peroxisome proliferator-activated receptor coactivator-1alpha	tgacactgtggcagattgttatt	aacttcacagcatcttcaattgtatt	NM_031347
**SIRT1**	Sirtuin 1	aaagggccaagcagagaga	gtaaatcacacggcgctctt	NM_001107627.1
**TOMM20**	Translocase of outer mitochondrial membrane 20	cacctgacaaatgcaatcgct	aacactggtggtggaagagtc	NM_152935.1

Table S1. Primer sequences for real-time qPCR.

Mitochondrial respirometry

Rats were killed by rapid decapitation on P45, directly at the end of the play fighting test. The nucleus accumbens and amygdala were rapidly dissected out, weighed and placed in a petri dish on ice with 2 ml of relaxing solution (2.8 mM Ca2K2EGTA, 7.2 mM K2EGTA, 5.8 mM ATP, 6.6 mM MgCl2, 20 mM taurine, 15 mM sodium phosphocreatine, 20 mM imidazole, 0.5 mM dithiotreitol and 50 mM ES, pH = 7.1) until further processing. Tissue samples were then gently homogenized in ice-cold respirometry medium (Oroboros Mir06: 0.5 mM EGTA, 3 mM MgCl2, 60 mM potassium lactobionate, 20 mM taurine, 10 mM KH2PO4, 20 mM HEPES, 110 mM sucrose, 0.1% (w/v) BSA, pH = 7.1 and 2800 units/mg catalase) with an Eppendorf pestle. Then, 2 mg of tissue were used to measure mitochondrial respiration rates at 37°C using high resolution respirometry (Oroboros Oxygraph 2K, Oroboros Instruments, Innsbruck, Austria), as previously described for other tissues (29). A multisubstrate protocol was used to sequentially exploring the various components of mitochondrial respiratory capacity, as previously described for the NAc and BLA (30,31). Briefly, to measure the respiration due to oxidative phosphorylation, we added substrates for the activation of specific complexes. Thus, oxygen flux due to complex I activity (Complex I) was quantified by the addition of ADP (5 mM) to a mixture of malate (2mM), pyruvate (10mM) and glutamate (20mM), followed by the addition of succinate (10 mM) to subsequently stimulate complex II (Complex I + II). We then uncoupled

respiration to examine the maximal capacity of the electron transport system (ETS) using the protonophore, carbonylcyanide 4 (trifluoromethoxy) phenylhydrazone (FCCP). We then examined consumption in the uncoupled state due solely to the activity of complex II by inhibiting complex I with the addition of rotenone (0.1µM; ETS CII). Finally, electron transport through complex III was inhibited by adding antimycin (2 µM) to obtain the level of residual oxygen consumption (ROX) due to oxidating side reactions outside of mitochondrial respiration. The O2 flux obtained in each step of the protocol was normalized by the wet weight of the tissue sample used for the analysis and

Supplementary Results

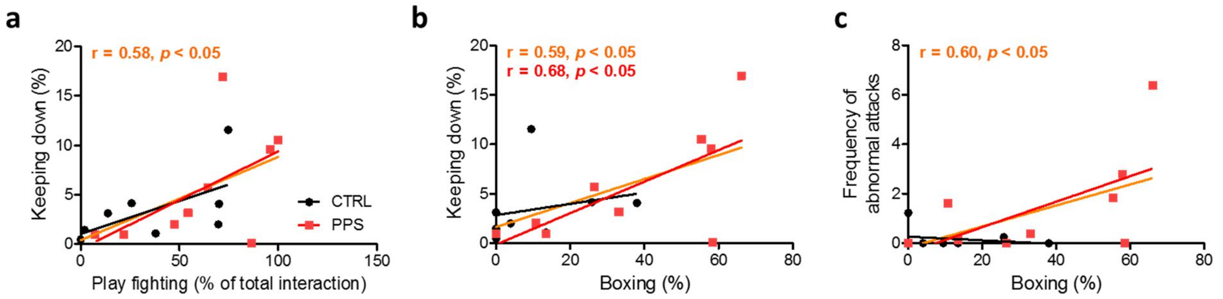

Figure S1 Correlations between play fighting and resident-intruder test parameters. Play fighting (**a,** r_{16} = 0.58, $p < 0.05$) and boxing percentage (**b,** r_{16} = 0.59, $p < 0.05$) correlated positively with keeping down duration in the resident-intruder test, when considering all data together, but also taking into consideration only PPS rats for boxing percentage **(b,** r_{14} = 0.68, $p < 0.05$). The boxing percentage also correlated positively with the frequency of abnormal attacks **(c,** r_{16} = 0.60, $p < 0.05$, all data together). *N:* CTRL= 8 and PPS = 9. Results are expressed as mean ± SEM.

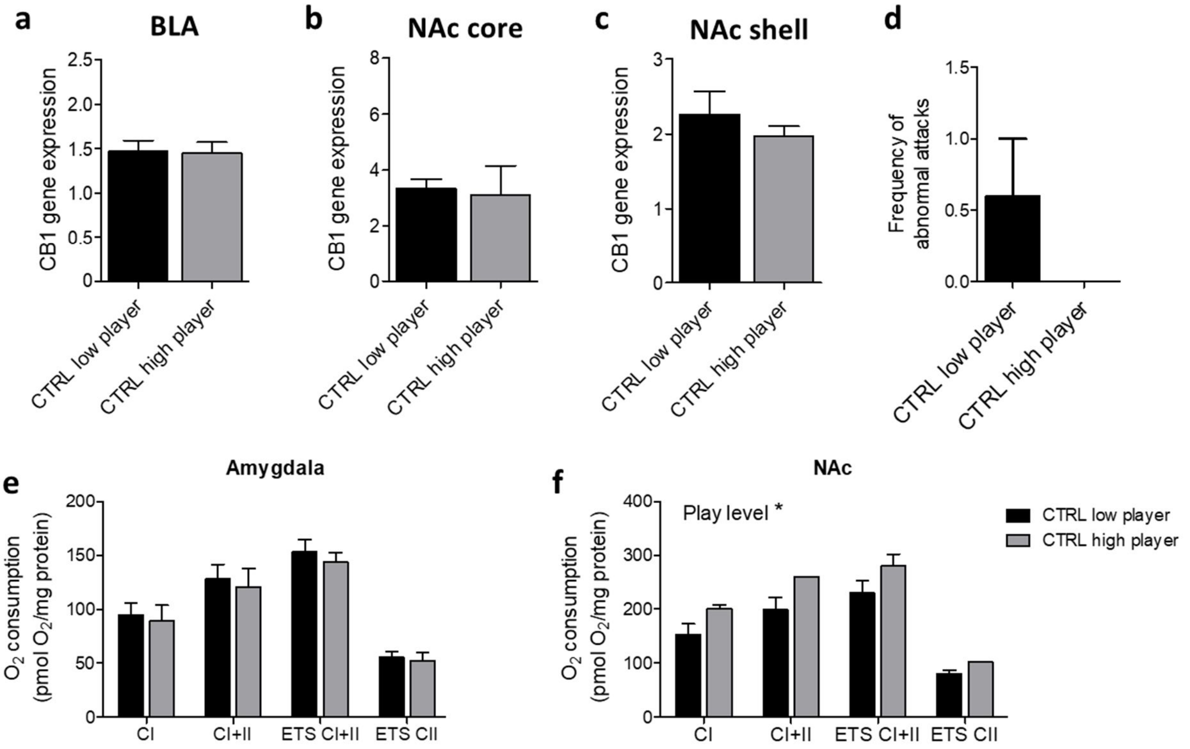

Fig S2 Analysis on CTRL rats according to the levels of play fighting. The analysis according to the levels of play fighting did not reveal differences in the expression of CB1 gene in the BLA (**a,** t_8 = 0.47, n.s.), NAc core (**b,** t_8 = 0.26, n.s.) or shell (**c,** t_{10} = 0.57, n.s.). The frequency of abnormal attacks was similar between low and high play (**d,** U = 6.00, n.s.). Respiration analysis was not different either among groups in the amygdala (**e,** $F_{(1, 24)}$ = 0.40, n.s.) but a significant effect of play level was found in the NAc respiration (**f;** $F_{(1, 24)}$ = 6.37, $p < 0.05$). Note however that for respiration analysis, there were only 2 CTRL high player rats. *N*: CTRL low player = 7-8; CTRL high player = 3-4, except for respiration, where CTRL low player = 6 and CTRL high player = 2. Results are expressed as mean ± SEM.

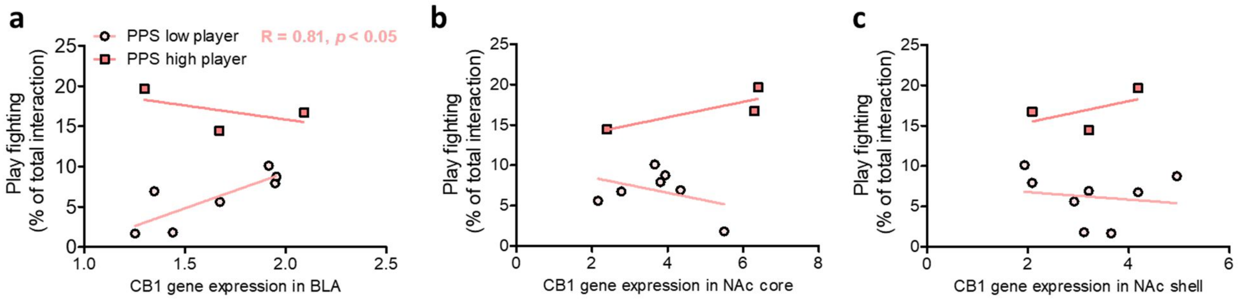

Figure S3 Correlations between play fighting and CB1 gene expression. Play fighting percentage in PPS low player correlated positively with CB1 expression in the BLA (**a,** $r_7 = 0.81$, $p < 0.05$) but not in the NAc core (**b,** $r_7 = -0.38$, n.s.) or shell (**c,** $r_8 = -0.15$, n.s.). *N:* PPS low player = 6-7, PPS high player = 3.

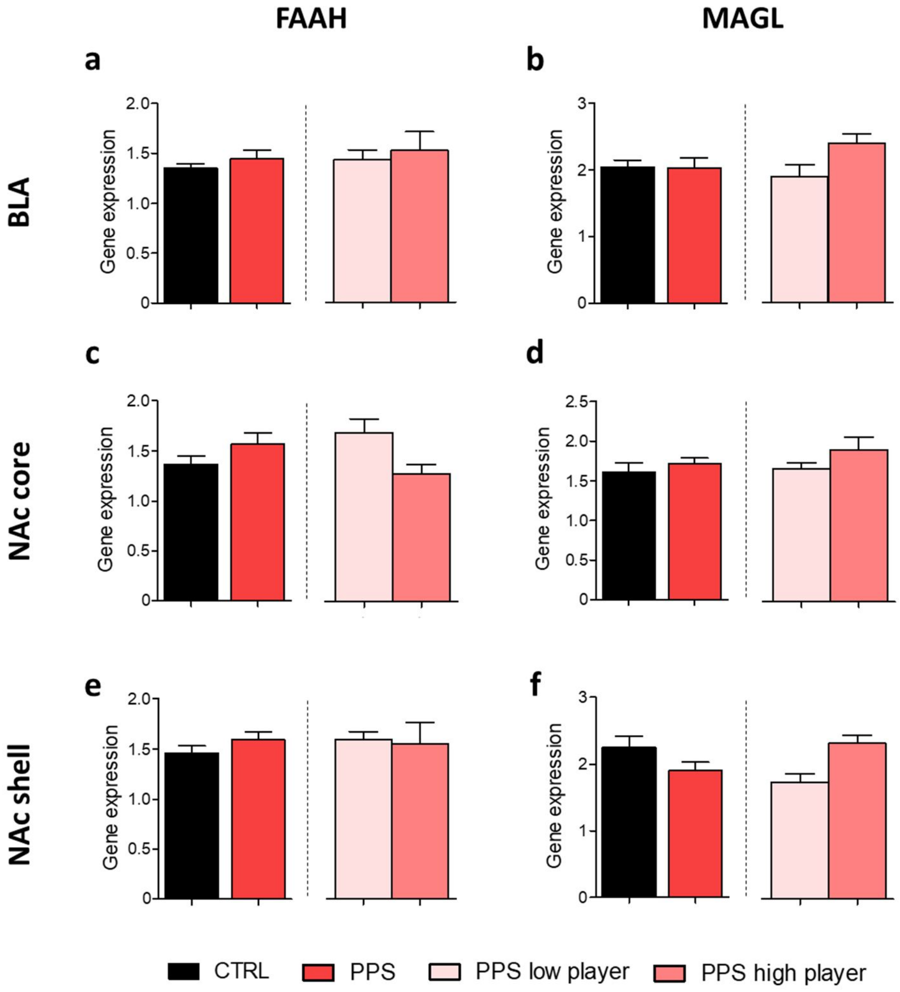

Figure S4 Gene expression analysis in the BLA (top), NAc core (middle) and NAc shell (bottom) following play fighting. Investigating further the potential role of the endocannabinoid system, we also evaluated the expression of the fatty acid hydrolase (FAAH) and monoacylglycerol lipase (MAGL), two catabolic enzymes of the endogenous ligands AEA and 2-AG. No significant difference was observed in FAAH gene expression in any of these brain region (**a,** BLA: t_{20} = 0.99, n.s.; **c,** NAc core: t_{21} = 1.43, n.s., **e,** NAc shell: t_{21} = 1.30, n.s.; even when the two PPS groups were analyzed separately: all $p > 0.05$). Expression levels of MAGL was similar between groups in the BLA (**b,** t_{20} = 0.07, n.s.) and in the NAc core (**d,** t_{21} = 0.73, n.s), even when the two PPS groups were analyzed separately (all $p > 0.05$). In the NAc shell, expression of MAGL did not differ between CTRL and PPS rats (**f,** t_{21} = 1.62, n.s.). However, ANOVA for MAGL was significant ($H(3) = 6.81$, $p < 0.05$), but the tendency for the PPS low players to show lower levels than the other two groups did not reach statistical significance in *post hoc* tests **(f).** *N:* CTRL= 10-12 and PPS = 9-11; PPS low player = 8, PPS high player = 3. Results are expressed as mean ± SEM.

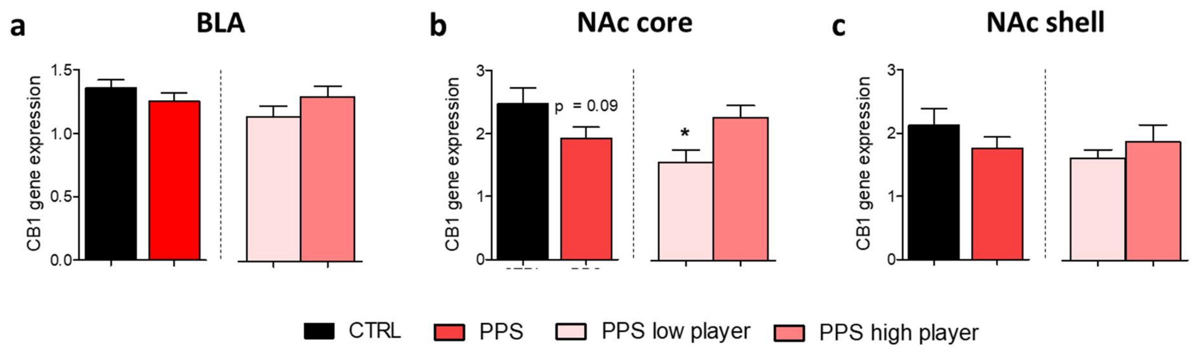

Figure S5 CB1 gene expression in the BLA, NAc core and shell at adulthood. The analysis of CB1 gene expression at adulthood did not reveal significant difference in the BLA (**a**, t_{15} = 1.12, n.s., even when just the two PPS groups were compared: all $p > 0.05$) and NAc shell (**c**, t_{14} = 1.17, n.s., even when just the two PPS groups were compared: all $p > 0.05$) between the groups. A tendency for decreased CB1 expression in PPS animals in the NAc core was observed (**b**, t_{14} = 1.84, p = 0.09) and analysis according to play levels showed a significant decrease in PPS low player compared to CTRL rats (**b**, H(3) = 7.60, $p > 0.05$, *post hoc* $p < 0.05$). *N*: CTRL = 7-8; PPS = 9, PPS low player = 3, PPS high player = 6. Results are expressed as mean ± SEM.

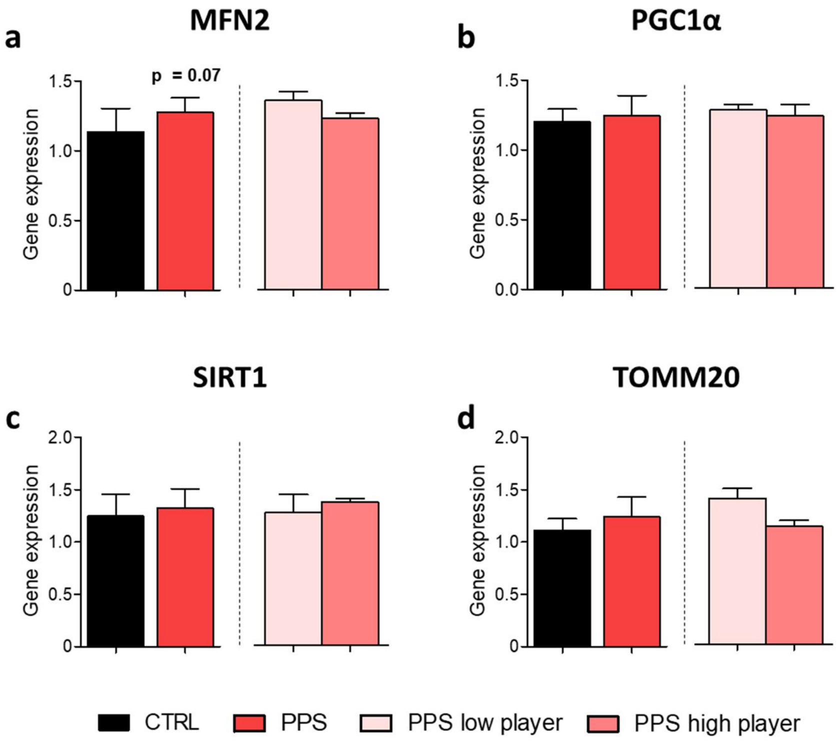

Figure S6 Gene expression analysis in the amygdala homogenates following mitochondrial respiration measurement. No significant differences were observed for the expression levels of PGC1α (**b,** t_{13} = 0.71, n.s), SIRT1 (**c,** t_{13} = 0.79, n.s) and TOMM20 (**d,** t_{12} = 1.52, n.s), even when the two PPS groups were analyzed separately (all $p > 0.05$). A trend toward increased expression of MFN2 in PPS animals was observed (**a,** t_{13} = 1.97, p = 0.07) but the stratification analysis did not reveal further significant difference (H(3) = 4.73, n.s.). *N:* CTRL= 8 and PPS = 8; PPS low player = 3, PPS high player = 5. Results are expressed as mean ± SEM.

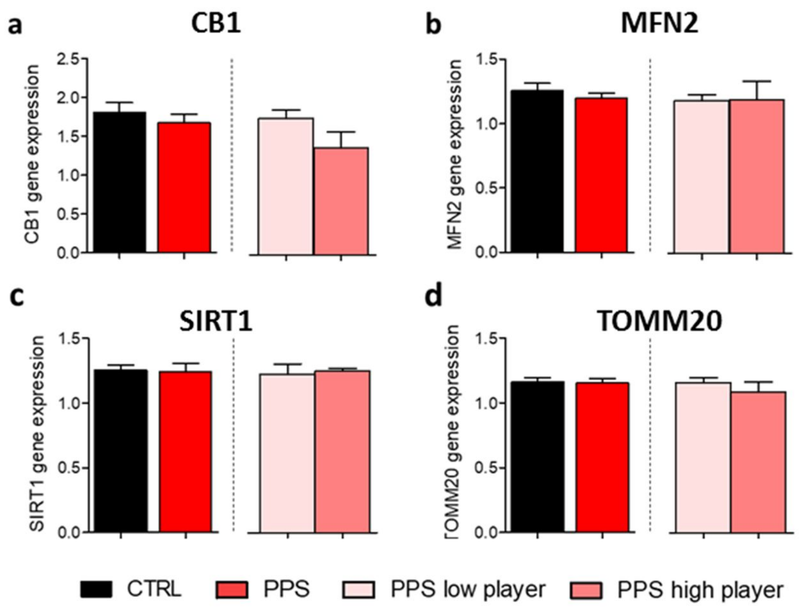

Figure S7 Expression analysis of CB1 and mitochondrial function-related genes in the CeA at peripuberty. Gene analyses in the CeA at peripuberty did not reveal significant difference between groups in the expression of CB1 (**a**, t_{17} = 0.81, n.s., even when just the two PPS groups were compared: all $p > 0.05$), MFN2 (**b**, t_{17} = 0.91, n.s., even when just the two PPS groups were compared: all $p > 0.05$), SIRT1 (**c**, t_{17} =0.18, n.s., even when just the two PPS groups were compared: all $p > 0.05$) and TOMM20 (**d**, t_{16} = 0.22, n.s., even when just the two PPS groups were compared: all $p > 0.05$). *N*: CTRL = 7-8; PPS = 9, PPS low player = 7, PPS high player = 2. Results are expressed as mean ± SEM.

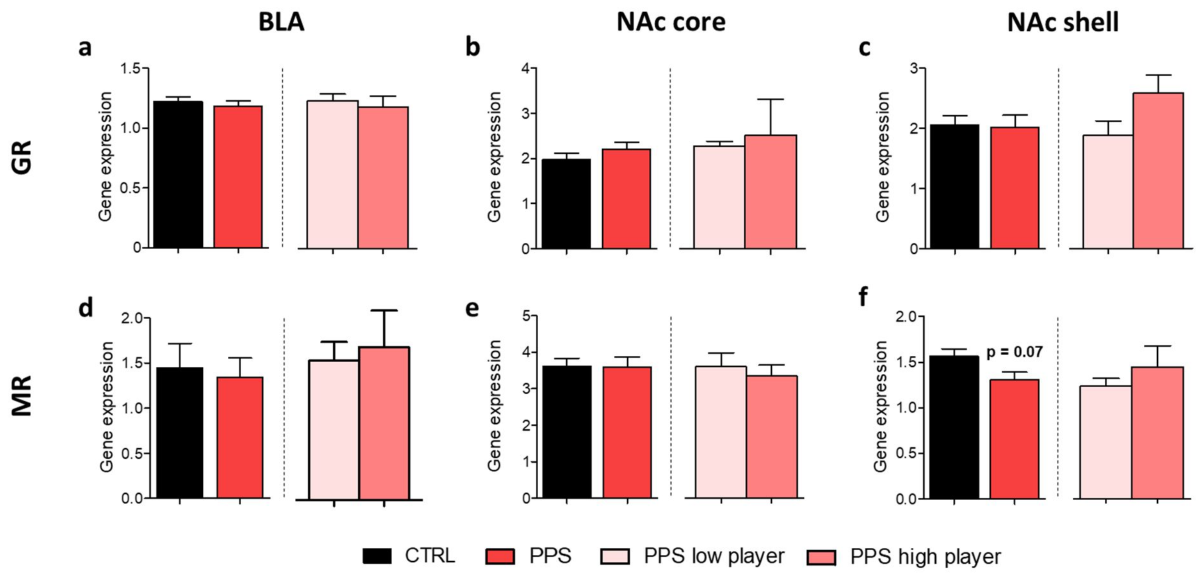

Figure S8 Expression analysis of GR and MR in the BLA, NAc core and shell at peripuberty. The analysis of GR expression at peripuberty when comparing PPS and control animals, as well as PPS high and low players, did not reveal significant difference in the BLA (a, *U* = 51.00, n.s., even when just the two PPS groups were compared: all $p > 0.05$), NAc core (b, $t_{18} = 1.20$, n.s., even when just the two PPS groups were compared: all $p > 0.05$) and NAc shell (c, $t_{21} = 0.19$, n.s., even when just the two PPS groups were compared: all $p > 0.05$). MR expression was also similar between groups in the BLA (d, $t_{21} = 1.03$, n.s., even when just the two PPS groups were compared: all $p > 0.05$) and NAc core (e, *U* = 34.00, n.s., even when just the two PPS groups were compared: all $p > 0.05$) A tendency for decreased MR expression in PPS animals in the NAc shell was observed (f, *U* = 32.00, p = 0.07) but no significant change was found when the two PPS groups were compared (all $p > 0.05$). *N*: CTRL = 10-12; PPS = 9-11, PPS low player = 3, PPS high player = 6-8. Results are expressed as mean ± SEM.

Supplementary References

1. Moy SS, Nadler JJ, Perez A, Barbaro RP, Johns JM, Magnuson TR, et al. Sociability and preference for social novelty in five inbred strains: an approach to assess autistic-like behavior in mice. Genes Brain Behav. 2004 Oct 1;3(5):287–302.

2. Porsolt RD, Anton G, Blavet N, Jalfre M. Behavioural despair in rats: a new model sensitive to antidepressant treatments. Eur J Pharmacol. 1978 Feb 15;47(4):379–91.
